# Fatal toxic shock syndrome following tattooing

**DOI:** 10.1007/s12024-025-01068-7

**Published:** 2025-08-13

**Authors:** Kristin Schreiner, Marek Balikowski

**Affiliations:** Institute of Legal Medicine and Traffic Medicine, Heidelberg University Hospital, Im Neuenheimer Feld 220/1, 69120 Heidelberg, Germany

**Keywords:** Postmortem diagnosis, *Staphylococcus aureus*, Tattooing, Sudden death, Sepsis

## Abstract

**Background:**

Toxic shock syndrome (TSS) is a rare but potentially fatal condition caused by toxin-producing strains of *Staphylococcus aureus*. While typically associated with menstruation or postoperative complications, non-menstrual forms related to skin trauma, including tattooing, are increasingly recognized.

**Case presentation:**

We report the sudden death of a woman in her early thirties who had experienced dizziness and circulatory instability in the days preceding death. She had recently undergone multiple tattoo sessions for a full-arm (“sleeve”) tattoo. At medicolegal external examination, no traumatic injuries were found apart from a recent tattoo-associated skin lesion but the postmortem rectal temperature was elevated at 41.1 °C. Autopsy revealed white mucous content in the airways and widespread coagulated blood in major vessels, but no morphological cause of death. Postmortem blood cultures grew *Staphylococcus aureus*, and PCR confirmed the presence of the toxic shock syndrome toxin-1 (TSST-1) gene. Histological examination of the tattooed skin demonstrated granulocytic infiltration consistent with acute local inflammation. Findings were interpreted as consistent with fulminant TSS, with the tattooed skin as a plausible bacterial entry site.

**Conclusion:**

This case emphasizes the need to consider TSS as a differential diagnosis in sudden deaths with systemic inflammatory features, particularly when recent skin trauma is present. Postmortem microbiological and molecular diagnostics are crucial in establishing this rare diagnosis.

## Introduction

Toxic shock syndrome (TSS) is a fulminant, toxin-mediated multisystem illness primarily caused by toxigenic strains of *Staphylococcus aureus* or *Streptococcus pyogenes*. While originally described in association with tampon use during menstruation, it is now recognized that up to 50% of cases occur in non-menstrual contexts, including postoperative infections, burns, soft tissue trauma, and even minor skin breaches [1, 2].

Central to the pathophysiology of TSS is the release of superantigenic exotoxins such as toxic shock syndrome toxin-1 (TSST-1). Unlike conventional antigens, superantigens bypass normal antigen processing and bind directly to MHC class II molecules and T-cell receptors, resulting in non-specific activation of up to 20% of the body’s T lymphocytes. This leads to a massive cytokine release (“cytokine storm”) with subsequent vascular leakage, hypotension, and subsequent multiorgan failure [3, 4].

Tattooing, though widely practiced and generally safe under hygienic conditions, can serve as an entry portal for bacteria through breaches in the skin barrier. In rare cases, tattoo-associated infections may progress beyond localized inflammation to systemic disease. A published case describes a 26-year-old man who developed classic TSS days after tattooing, with positive *S. aureus* cultures and a fulminant clinical course [5]. While most such infections remain localized, this case demonstrates the potential for tattooing to facilitate invasive toxigenic infections under certain conditions.

From a forensic medical perspective, TSS poses a considerable diagnostic challenge, particularly in postmortem settings, where classic clinical features like rash or desquamation may be absent or nonspecific. Hyperthermia, intravascular coagulation, and signs of systemic inflammation may be present but are not pathognomonic. In cases where the body is found shortly after death, elevated postmortem core temperature may reflect ante-mortem hyperthermia and support the suspicion of TSS. Especially in cases where the body is found soon after death, postmortem microbiological and molecular diagnostics (e.g., PCR for TSST-1 genes) may be crucial in clarifying the cause of sudden, unexplained deaths—particularly in otherwise healthy individuals with recent skin trauma or tattoos [6, 7].

Given the increasing prevalence of body modifications and the diagnostic ambiguity of TSS at autopsy, awareness of tattoo-related TSS as a differential diagnosis is essential for forensic pathologists.

### Case presentation

**Fig. 1:**
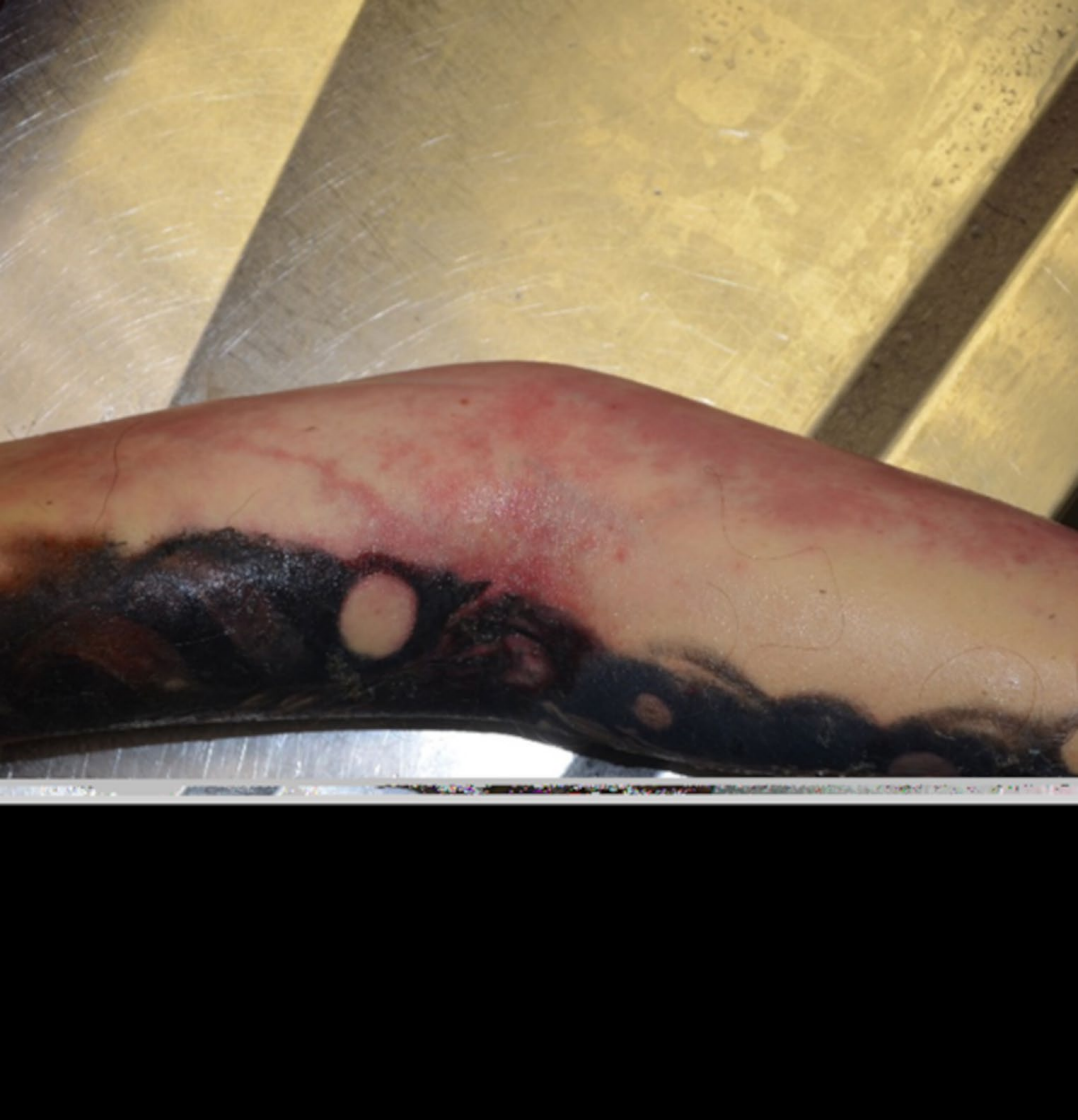
Clinical photograph of the decedent’s right arm with a large black-ink tattoo. The antecubital area shows marked red discoloration, without signs of necrosis or ulceration. No visible signs of skin breakdown or ulceration are present

In September 2024, the body of a woman in her early thirties was found in a private residence. According to background information, she had reported dizziness and circulatory instability in the days before she died. She had recently undergone multiple tattoo sessions as part of a full-arm (“sleeve”) tattoo. As shown in Fig. 1, the tattooed skin exhibited marked reddish discoloration—most pronounced in the antecubital region—without macroscopic signs of necrosis.

At medicolegal external on-site examination, no external injuries were noted aside from healing tattoos covering one entirety arm. Rectal core body temperature was recorded at 41.1 °C.

**Fig. 2:**
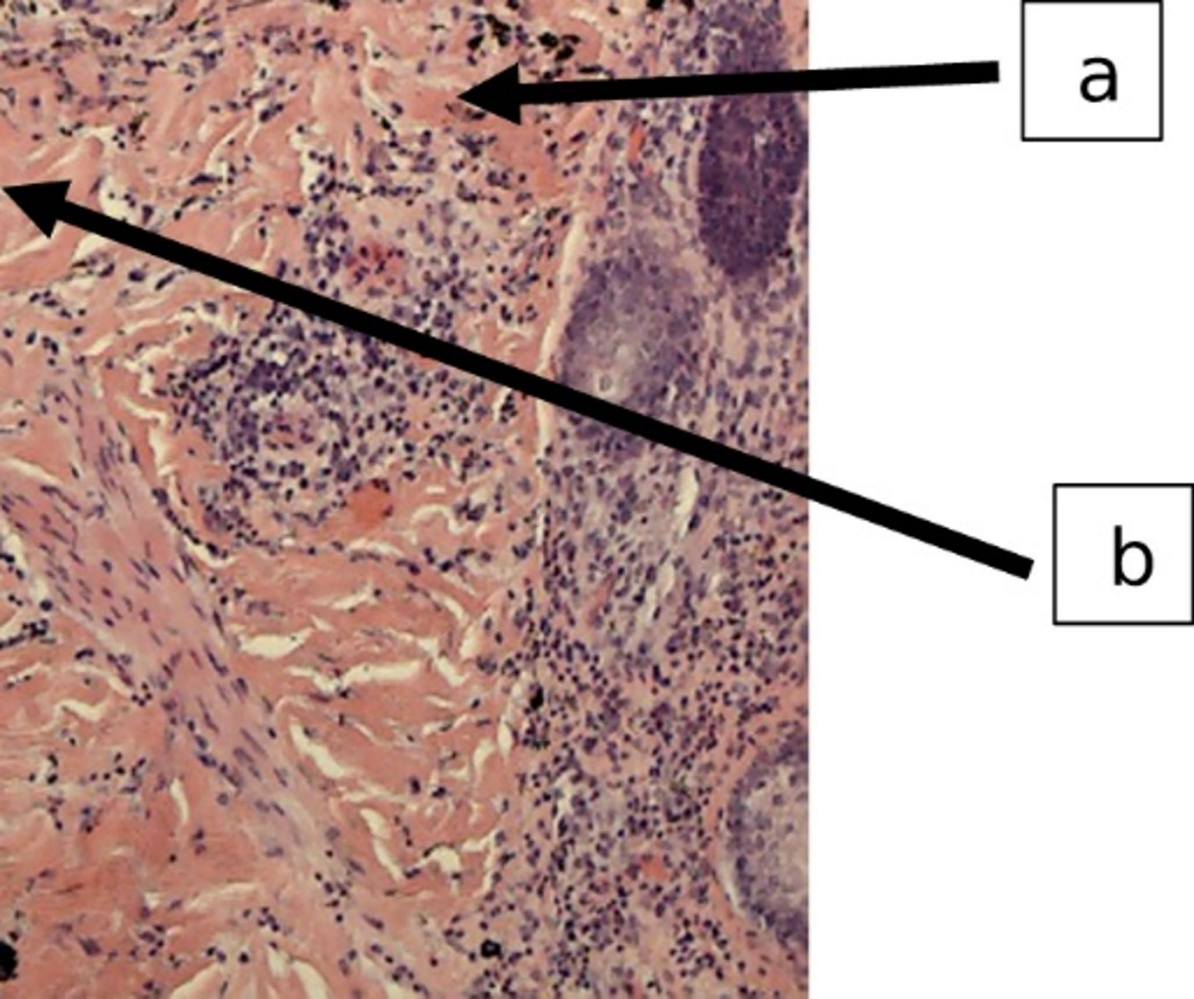
Histological section of tattooed skin stained with hematoxylin and eosin (HE), showing preserved epidermal architecture and dense black pigment deposits consistent with tattoo ink in the dermis (**a**). The dermis and adjacent subcutaneous tissue show a pronounced acute inflammatory infiltrate dominated by neutrophilic granulocytes (e.g. **b**). There was no evidence of necrosis, ulceration, or chronic inflammation

Autopsy revealed white, viscous material within the trachea and lower airways. Major vessels contained dense, coagulated blood. No evidence of mechanical trauma or natural disease was found. Microbiological analysis of postmortem femoral blood revealed *Staphylococcus aureus* growth. PCR testing confirmed the presence of the toxic shock syndrome toxin-1 (TSST-1) gene. Histological examination of the tattooed skin showed marked infiltration with granulocytes extending into the subcutaneous tissue, consistent with acute inflammation. Histological analysis revealed granulocytic infiltration and dermal pigment deposits (Fig. 2).

Additional organ histology was conducted. The brain, pancreas, spleen, and renal structures appeared unremarkable. The adrenal gland displayed a small cortical adenoma without signs of malignancy or systemic involvement. Cardiac tissue showed mild nuclear irregularities in the right ventricle but preserved architecture without evidence of myocarditis or inflammatory infiltration. Lung sections exhibited alveolar wall destruction, congestion, and focal edema but no signs of inflammation. The liver showed mild portal fibrosis and focal macrovesicular steatosis, but no necrosis or significant inflammation. Kidney tissue revealed nephrosclerosis.

The findings were consistent with fulminant toxic shock syndrome. A possible association with bacterial entry via the recently tattooed skin was considered.

## Discussion

Toxic shock syndrome is a rare, toxin-mediated condition that can progress rapidly to multiorgan failure and death. While most commonly associated with tampon use, an increasing number of non-menstrual cases have been described in association with soft tissue infections, minor trauma, and postoperative complications [1, 2]. Tattooing, which entails repeated skin penetration and induces a local inflammatory response, inherently breaches the skin barrier and may facilitate the entry of toxigenic *Staphylococcus aureus*, especially when hygienic standards are inadequate. In the present case, no other entry point was identified aside from the recently tattooed skin.

To date, only a few case reports have described TSS following tattooing. Jeong et al. reported a case of a 26-year-old man who developed high fever, hypotension, and a diffuse rash after a tattoo session. Culture of the tattooed skin yielded *S. aureus*, and the patient recovered with supportive therapy and antibiotics [3]. In contrast, the present case represents a fatal course in a previously healthy adult, highlighting the potential severity of this condition even in non-hospitalized individuals.

The pathogenic mechanism of TSS is mediated by superantigens such as toxic shock syndrome toxin-1 (TSST-1), which bypass normal antigen processing and lead to non-specific activation of a large proportion of T lymphocytes. This results in massive cytokine release, capillary leakage, and rapid hemodynamic deterioration [4, 5]. This finding is consistent with the proposed mechanism of superantigen-mediated immune activation underlying toxic shock syndrome. Moreover, the macroscopic finding of dense, coagulated blood in large vessels may reflect a hypercoagulable state, which has been reported in association with TSS-related systemic inflammation and endothelial injury [6].

Histological analysis of the tattooed skin revealed marked granulocytic infiltration, consistent with an acute inflammatory process. While histological analysis was limited to selected tissues, including the skin and major internal organs, no signs of chronic disease or alternative infectious entry sites were identified in this case, supporting the assumption of localized skin-associated entry. No evidence of chronic disease or other primary infection foci was found.

From a forensic perspective, the diagnosis of TSS post mortem poses significant challenges. The clinical hallmarks—fever, rash, hypotension—may be absent or unrecognizable after death. Macroscopic findings can be nonspecific, and standard autopsy may not reveal a cause of death. In such cases, microbiological cultures and molecular analyses are essential tools for postmortem investigation. Detection of TSST-1 and histological confirmation of acute inflammation in suspected entry sites can support the diagnosis of TSS and establish a plausible causal link [7, 8]. The value of postmortem microbiological diagnostics has been extensively discussed by Tsokos et al., who emphasize the importance of proper sampling technique, timing, and contextual interpretation in distinguishing ante- from postmortem bacterial growth [9].

## Conclusion

This case illustrates that toxic shock syndrome can occur in association with tattooing, even in healthy adults. Postmortem diagnosis requires a high index of suspicion and must rely on microbiological, molecular, and histological findings to support the identification of TSS in otherwise unexplained deaths.

## Data Availability

Data available upon request.

## Declarations

### Clinical trial number

N/A.
